# Supplementary material for: Redistribution and Activation of CD16brightCD56dim NK Cell Subset to Fight against Omicron Subvariant BA.2 after COVID-19 Vaccination
Source: Microorganisms. 2023 Apr 3;11(4):940. doi: 10.3390/microorganisms11040940 (PMC10145754; doi:10.3390/microorganisms11040940)
Supplement: Supplementary file 1 [file microorganisms-11-00940-s001.zip › microorganisms-2241054-supplementary.pdf]

Supplementary Material

Table S1. Comparison of the lymphocyte subsets and cytokines between the unvaccinated and vaccinated groups on hospital admission.

Characteristic	Normal range	Unvaccinated group (n=102)	Vaccinated group (n=406)	*p*−value
Lymphocyte, median (IQR*), cells/μL	1530−3700	1389.50 (935.25−2080.50)	1400.00 (963.00−1887.00)	0.798
CD3+, median (IQR), cells/μL	955−2860	907.19 (516.25−1484.33)	930.94 (641.84−1314.86)	0.283
CD3+CD4+, median (IQR), cells/μL	550−1440	515.23 (288.45−833.94)	570.95 (369.60−810.81)	0.071
CD3+CD8+, median (IQR), cells/μL	320−1250	286.04 (172.57−487.04)	300.16 (187.97−424.30)	0.276
CD3-CD56+CD16+, median (IQR), cells/μL	150−1100	178.35 (100.43−330.00)	244.26 (139.83−364.03)	0.001
CD19+, median (IQR), cells/μL	90−560	128.21 (47.66−321.83)	131.60 (80.13−218.68)	0.435
CD3+, median (IQR),%	50−84	70.03 (59.98−75.60)	70.00 (63.38−75.94)	0.474
CD3+CD4+, median (IQR), %	27−51	39.13 (32.06−45.08)	40.85 (35.42−48.25)	0.071
CD3+CD8+, median (IQR), %	15−44	20.11 (15.40−27.65)	21.49 (16.50−26.90)	0.400
CD3-CD56+CD16+, median (IQR), %	7−40	13.20 (9.31−20.48)	17.51 (12.36−24.69)	＜0.000
CD19+, median (IQR), %	5−18	10.83 (6.63−17.11)	9.90 (7.18−13.32)	0.308
CD4+/CD8+, median (IQR)	0.71−2.78	1.86 (1.48−2.67)	1.94 (1.42−2.65)	0.787
IFN−γ, median (IQR), pg/mL	0−4.43	1.63 (1.49−1.99)	1.77 (1.55−2.85)	0.001
IL−1β, median (IQR), pg/mL	0−3.40	3.50 (2.98−4.20)	4.39 (3.33−8.74)	＜0.000
IL−2, median (IQR), pg/mL	0−6.64	2.79 (2.52−3.34)	3.19 (2.73−5.00)	＜0.000
IL−4, median (IQR), pg/mL	0−4.19	2.52 (2.16−3.03)	2.91 (2.34−4.69)	0.001
IL−5, median (IQR), pg/mL	0−4.15	2.92 (2.49−3.66)	3.28 (2.74−5.05)	＜0.000
IL−6, median (IQR), pg/mL	0−11.09	3.22 (2.72−4.32)	3.65 (2.82−6.25)	0.024
IL−8, median (IQR), pg/mL	0−15.71	6.98 (4.69−10.80)	7.42 (4.9−11.62)	0.755
IL−10, median (IQR), pg/mL	0−4.5	4.08 (3.55−4.95)	4.66 (3.90−8.18)	＜0.000
IL−12p70, median (IQR), pg/mL	0−10.18	1.84 (1.70−2.03)	1.92 (1.70−2.03)	0.380
IL−17A, median (IQR), pg/mL	0−4.74	3.22 (2.73−3.76)	3.54 (2.92−4.95)	0.003
IL−17F, median (IQR), pg/mL	0−4.66	1.45 (1.32−1.64)	1.51 (1.32−1.64)	0.079
IL−22, median (IQR), pg/mL	0−3.64	2.28 (1.97−2.91)	2.59 (2.11−3.48)	0.002
TNF−α, median (IQR), pg/mL	0−4.5	4.61 (3.86−5.86)	5.30 (4.18−9.00)	0.002
TNF−β, median (IQR), pg/mL	0−2.54	1.88 (1.63−2.18)	1.98 (1.76−2.38)	0.001

*IQR: interquartile range

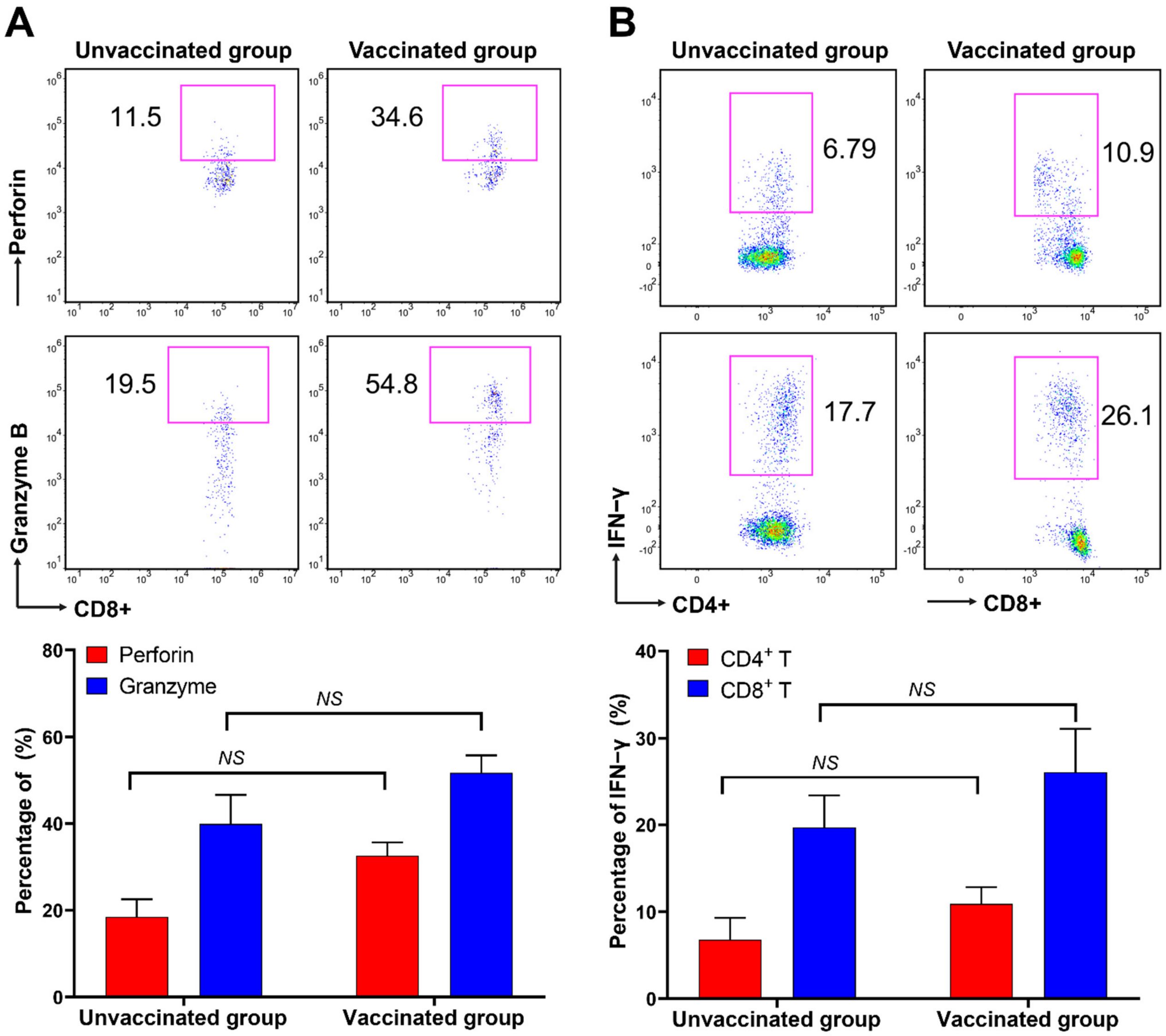

Figure S1. Comparison of perforin, granzyme B, and IFN−γ expression on T cells between unvaccinated and vaccinated groups. (A) The expression of perforin and granzyme B on CD8+ T cell subsets was detected using flow cytometry. Unvaccinated group, n = 18; Vaccinated group, n = 50, respectively. (B) The expression of IFN−γ on CD4+ and CD8+ T cell subsets were evaluated using flow cytometry. Unvaccinated group, n = 11; Vaccinated group, n = 45, respectively. All *p* values were two−tailed, and differences with $p < 0.05$ were considered statistically significant. ns, not significant.
